# Biallelic *CRELD1* variants cause severe muscle weakness and infantile epilepsy

**DOI:** 10.1093/braincomms/fcaf326

**Published:** 2025-09-03

**Authors:** Manuela D'Alessandro, Daniel Bamborschke, Margret H Bülow, Özkan Özdemir, Hülya-Sevcan Daimagüler, Verena Brümmer, Nicole Kucharowski, Julia Sellin, Anna Brunn, Martina Deckert, Gilbert Wunderlich, Friederike Koerber, Jean-Louis Bessereau, Sebahattin Cirak

**Affiliations:** Univ Lyon, University Claude Bernard Lyon 1, MeLiS, CNRS UMR 5284, INSERM U 1314, Lyon 69008, France; Center for Molecular Medicine Cologne (CMMC), Faculty of Medicine and University Hospital Cologne, University of Cologne, Cologne 50931, Germany; Department of Pediatrics, Faculty of Medicine and University Hospital Cologne, University of Cologne, Cologne 50937, Germany; Life and Medical Sciences Institute, University of Bonn, Bonn 53115, Germany; Klinik für Herzchirurgie, Cure3D lab, Department Membrane Contact Sites, Universitätsklinikum Düsseldorf, Düsseldorf 40225, Germany; Center for Molecular Medicine Cologne (CMMC), Faculty of Medicine and University Hospital Cologne, University of Cologne, Cologne 50931, Germany; Department of Pediatrics, Faculty of Medicine and University Hospital Cologne, University of Cologne, Cologne 50937, Germany; Center for Molecular Medicine Cologne (CMMC), Faculty of Medicine and University Hospital Cologne, University of Cologne, Cologne 50931, Germany; Department of Pediatrics, Faculty of Medicine and University Hospital Cologne, University of Cologne, Cologne 50937, Germany; Department of Pediatrics, Faculty of Medicine and University Hospital Cologne, University of Cologne, Cologne 50937, Germany; Life and Medical Sciences Institute, University of Bonn, Bonn 53115, Germany; Klinik für Herzchirurgie, Cure3D lab, Department Membrane Contact Sites, Universitätsklinikum Düsseldorf, Düsseldorf 40225, Germany; Institute for Digitalization and General Medicine, University Hospital RWTH Aachen, Aachen 52064, Germany; Institute of Neuropathology, Faculty of Medicine and University Hospital Cologne, University of Cologne, Cologne 50937, Germany; Institute of Neuropathology, Faculty of Medicine and University Hospital Cologne, University of Cologne, Cologne 50937, Germany; Department of Neurology, Faculty of Medicine and University Hospital Cologne, University of Cologne, Cologne 50937, Germany; Center for Rare Diseases, Faculty of Medicine and University Hospital Cologne, University of Cologne, Cologne 50937, Germany; Department of Pediatric Radiology, Faculty of Medicine and University Hospital Cologne, University of Cologne, Cologne 50937, Germany; Univ Lyon, University Claude Bernard Lyon 1, MeLiS, CNRS UMR 5284, INSERM U 1314, Lyon 69008, France; Center for Molecular Medicine Cologne (CMMC), Faculty of Medicine and University Hospital Cologne, University of Cologne, Cologne 50931, Germany; Division of Pediatric Neurology, Metabolics and Social Pediatrics, Department of Pediatrics and Adolescent Medicine, Ulm University Medical Center, Ulm University, Ulm 89075, Germany

**Keywords:** CRELD1, nicotinic acetylcholine receptor, arthrogryposis, epilepsy

## Abstract

Nicotinic acetylcholine receptors are widely expressed in the peripheral and central nervous systems. Mutations in acetylcholine receptor-subunit genes have been associated with neuromuscular diseases, such as arthrogryposis multiplex congenita (AMC) and epilepsy. We report a patient with arthrogryposis, severe muscle weakness and neurodevelopmental delay. During his first year of life, he developed therapy-refractory epilepsy. Using whole-exome sequencing, we identified the compound pathogenic variants c. 875G>A (p. Cys292Tyr) and c. 959delA (p. Gln320Argfs*25) in the cysteine-rich with epidermal growth factor-like domain protein 1 gene (*CRELD1*, NM_001077415.3). Recently, functional studies have shown that CRELD1 is a membrane-associated endoplasmic reticulum-resident protein disulphide isomerase that acts as a maturation enhancer of AChR biogenesis, thereby controlling the abundance of functional receptors at the cell surface. To test pathogenicity, we took advantage of the genetics and extremely rapid genome editing in *Caenorhabditis elegans*. We were able to model these heterozygous variants and observed a decrease in AChRs at the neuromuscular junction. Hence, our study identifies compound heterozygous *CRELD1* variants responsible for a rare neurodevelopmental disorder characterized by arthrogryposis, muscle weakness and epilepsy.

## Teaser

D’Alessandro *et al.* reported that compound heterozygous *CRELD1* variants were identified in a patient with arthrogryposis, muscle weakness and epilepsy. Variant modelling in *C. elegans* causes reduced acetylcholine receptor levels at the neuromuscular junction, demonstrating variant pathogenicity and establishing *CRELD1* mutations as causative of this neurodevelopmental disorder.

## Graphical Abstract

**Graphical Abstract:**
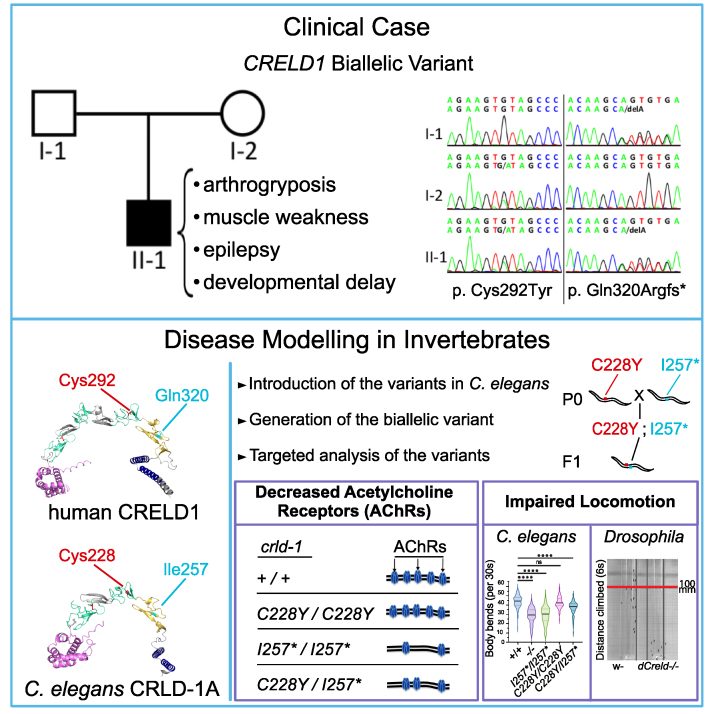

## Introduction

Nicotinic acetylcholine receptors (AChRs) are the major receptors at the neuromuscular junction (NMJ) and are widely expressed in the central nervous system.^1-4^ Pathogenic roles of AChRs have been identified in many diseases, affecting cell types ranging from muscle to neurons, with symptoms ranging from muscle weakness to arthrogryposis, epilepsy and neurogenerative diseases.^5-8^ AChRs are pentameric transmembrane proteins whose assembly appears to be slow and inefficient. In muscle cells, only 30% of synthesized α-subunits ultimately assemble into a mature pentamer^9^ and in neurons, a pool of immature receptors is readily detectable in intracellular compartments.^10^ The exact physiological role of the intracellular retention of AChRs is unclear, but emerging evidence suggests that it may contribute to disease mechanisms. Notably, a rare mutation in the human β4 subunit of the AChR has been linked to amyotrophic lateral sclerosis and has been shown to disrupt the trafficking of α4β4 receptors, leading to their accumulation in the endoplasmic reticulum (ER).^11^ Although genes involved in AChR biosynthesis and trafficking have been identified, the mechanisms by which defects in these genes and resulting disruptions in AChR trafficking contribute to human disease are largely unknown. An exception is a stillborn infant with an arthrogryposis phenotype carrying mutations in *UNC50*, a gene associated with AChR trafficking.^12^ Here, we report a patient with compound heterozygous *CRELD1* (NM_001077415.3) functional variants presenting with arthrogryposis and epilepsy, defining a mendelian recessive disease.

*CRELD1* is highly expressed in humans, particularly in the adult heart, brain and skeletal muscle.^13^ Missense variants in human *CRELD1* were initially associated with atrioventricular septal defects (AVSD),^14^ and allelic interactions between *CRELD1* and vascular endothelial growth factor-A have been shown to contribute to the development of AVSD.^15^ In mice, *Creld1*-null (*Creld1−/−*) embryos exhibit embryonic lethality by day 11.5, presenting with multiple abnormalities, including heart developmental defects.^16^ CRELD1 has also been identified as a critical regulator of immune system homeostasis in recent studies of gene expression variance in large human cohorts coupled with mouse models.^17^

CRELD1 proteins are evolutionarily conserved, and our work using *Caenorhabditis elegans* and mouse muscle cells has demonstrated that CRELD1 is a membrane-associated ER-resident protein disulphide isomerase (PDI). We have identified AChRs as a specific client of CRELD among other neurotransmitter receptors of the same protein superfamily that are not dependent on CRELD1, including type A GABA receptors. CRELD1 acts as a maturation enhancer of AChR biogenesis and controls their abundance at the cell surface.^18^ In a recent study, we have also described *Drosophila* Creld as a regulator of redox homeostasis, and *dCreld* mutants show severe locomotion defects.^19^

An international consortium has recently characterized 18 individuals with biallelic *CRELD1* variants who displayed a wide range of phenotypes, including developmental delay, early-onset epilepsy and hypotonia.^20^ However, because of the experimental strategy relying on the *Xenopus* tadpole, the study did not provide a comprehensive modelling and phenotypic characterization of the exact compound variants found in the patients. The work presented here uses the nematode *C. elegans* as a model organism to evaluate the functionality of *CRELD1* variants. It provides a pioneering approach to modelling a compound *CRELD1* variant mutation and assessing its pathogenicity, as few existing model systems are able to replicate this specific form of compound variant.

By taking advantage of both the *C. elegans* and *Drosophila* invertebrate model systems, we provide further evidence that a defect in CRELD1 function causes a neurodevelopmental disorder associated with epilepsy, arthrogryposis and muscle weakness, which may be partially explained by a defect in AChR biosynthesis.

## Materials and methods

### Genetic studies

Approval for the research performed in this publication has been granted by the ethics board of the University of Cologne [sign 19–1269]. After informed consent, peripheral venous blood was obtained from the index patient and both parents. Genomic DNA was extracted via QIAamp DNA Blood Mini Kit (Qiagen) following the manufacturers’ protocol. The index patient’s DNA was used for library preparation with the SeqCap EZ Human Exome Library v2.0 kit (Roche NimbleGen). Whole exome sequencing (WES) was performed on the Illumina HiSeq 2000 sequencing instrument with 2 × 100 bp as per the manufacturer’s recommendations (see Supplementary Methods). The Cologne Center for Genomics’ VARBANK pipeline v.2.12 (https://varbank.ccg.uni-koeln.de/) was used for annotation, pathogenicity prediction and variant filtering (see Supplementary Methods). Sanger sequencing of the *CRELD1* variants was performed for the index patient and his parents to analyze the variants’ co-segregation within the family (see Supplementary Methods).

### RNA isolation from muscle tissue and RT-qPCR/RT-PCR

We used commercial human tissue RNAs from TAKARA Scientific (Skeletal muscle Cat.# 636534; Fetal skeletal muscle cat.# 636747; Brain Cat.# 636530; and fetal brain Cat.# 636526) as well as patient and control muscle biopsies for identification of c.959delA (p.Gln320Argfs*25) associated expression change. The remaining tissue from the diagnostic muscle biopsy was used for further analysis after the patient consent of the index patient. The snap-frozen muscle sample was homogenized in 0.6 ml Trizol reagent (ThermoFisher Scientific, Cat.# 15596026) with QIAshredder (Qiagen, Cat.# 79656). RNA isolation was performed with the Qiagen RNeasy mini kit according to the manufacturer’s instructions (Qiagen Cat.# 74106). RNA purity, integrity and concentration were assessed via Nanodrop (ThermoFisher Scientific), Bioanalyzer (Agilent Technologies Inc.) and Qubit Fluorometer (ThermoFisher Scientific), respectively, for the isolated RNA samples. We performed cDNA synthesis with RNAs that had an RIN value higher than 7. SuperScript III reverse transcriptase (ThermoFisher Scientific, Cat.# 18080200) was used with 8 ng starting RNA for each sample according to manufacturer’s instructions with oligoDT primers. qPCR reactions were carried out via GoTaq qPCR Master Mix (Promega, Cat.# A6002). Additionally, we used log10 serial dilutions as an internal control of our PCR reactions. 2^−ΔΔCT^ method was used to calculate the relative fold changes between the patient and control samples with *GAPDH* as the reference gene.

To evaluate the effect of the c.875G>A (p.Cys292Tyr) variant located in Exon 8 on splicing, RT-PCR was performed with a pair of primers (F-GTGAAGGAGAAGGGACACGA; R-CTTGTTCTCTCCCGGACACA) spanning exons 5 to 9. The products were Sanger sequenced, and size was evaluated by agarose gel electrophoresis.

### *Caenorhabditis elegans* strains

#### Strains and genetics

*C. elegans* strains were maintained following established protocols^21^ and kept at 20°C. This study employed the following mutations: LG IV: *crld-1(kr297, kr308, kr586, kr587, kr871)*. Details of the strains, expression constructs, transgenic animals and knock-in worm generation can be found in Supplementary Table 1.

#### List of strains

The following mutant alleles and transgenes were used in this study: LGI: *krSi144(unc-17-cla1-BFP), kr208(unc-29::TagRFP-T)*; LGIV: *kr297(crld-1::HySOG), kr298(crld-1::GFP), kr308(crld-1a::GFP), kr586(crld-1a[C187Y]), kr587(crld-1a[C228Y]), kr871(crld-1[*I257Rfs*25*])*. A comprehensive list of all strains and their corresponding genotypes used in this study is provided in Supplementary Table 1.

The three patient variants Cys192Tyr, Cys292Tyr and Gln320Argfs25 correspond to the *C. elegans* mutations C187Y, C228Y and I257Rfs*25, respectively, as detailed in the Results section: ‘Generation and characterization of a CRELD1 knock-in *C. elegans* model using CRISPR/Cas9.’ Multiple CRISPR lines were generated in different gfp-*crld-1* backgrounds, and as shown in Supplementary Fig. 1, no significant differences were observed among independent CRISPR lines carrying the same mutation. Alleles *kr298*, *kr308*, *kr297* and *kr208* were previously described and characterized.^18^ In the results, control strains marked with a ‘+’ correspond to *gfp-crld-1* knock ins alone or with *unc-29-rfp* in the background, while the *crld-1* null mutant (*crld-1-HySOG*) is indicated with a ‘−’. The allele *krSi144(unc-17::cla-1-BFP*) is a single-copy insertion transgene used as a marker of cholinergic presynaptic boutons, which enabled the selection of the F1 progeny carrying compound heterozygous *crld-1* mutations in crosses between C228Y and I257Rfs*25 or the null mutant (*crld-1::HySOG)*.

#### Generation of single-copy insertion mutant allele by CRISPR/Cas9 technology

The *crld-1a* knock-ins containing the C187Y and the C228Y point mutations were created through CRISPR/*Cas9*-based homologous recombination by injecting *Cas9* ribonucleoprotein complexes into the syncytial gonad of 1-day-old *crld-1a(kr308::GFP)* hermaphrodites together with single-strand DNA oligonucleotide repair templates following the protocol described in Soussia *et al.* 2019.^22^ Engineered worms were identified by PCR and confirmed by Sanger sequencing. A similar strategy was used to generate the I257Rfs*25 mutation. For details on genome engineering, reagents see Supplementary Table 2.

#### Levamisole test

Assays for levamisole sensitivity after overnight exposure were assessed using previously established methods.^18^ Tetramisole hydrochloride (Sigma-Aldrich) was prepared in water and incorporated into nematode growth medium agar, pre-equilibrated to 55°C, at final concentrations of 1 mM or 0.6 mM immediately prior to plate pouring. The levamisole-supplemented plates were subsequently seeded with OP50 *Escherichia coli*. Young adult worms were transferred onto the levamisole-containing plates, incubated overnight at 20°C, and assessed for paralysis the following day.

#### Thrashing assay

Body bends frequency (BBF) measurements were performed.^23^ In brief, 1-day-old adult worms were carefully placed into wells of a 12-well cell culture plate (ten worms per well), each containing 1.5 mL of 2% agarose in M9 buffer and an additional 2 mL of M9 buffer. Two minutes post-transfer, worm movement was recorded for 30 s. BBF was quantified using the open-source wrMTrck plugin within Fiji (ImageJ v2.0.0) software.

#### Microscopy and fluorescence quantification

Animals were imaged at the young adult stage (24 h post L4 larval stage), live hermaphrodites were mounted on 2% agarose (w/v in water) dry pads immersed in 5% poly-lysine beads diluted in M9 buffer^24^ Confocal fluorescence imaging was performed using an Andor spinning disk confocal system (Oxford Instruments) mounted on a Nikon IX86 microscope (Olympus), equipped with a 60×/1.2 NA oil immersion objective and an Evolve EMCCD camera. Image acquisition was carried out using Andor’s iQ 3.4.1 software. For each worm, a z-stack of the ventral nerve cord (VNC) was captured at 0.2 µm intervals around the mid-body region, anterior to the vulva. Imaging parameters were kept constant across all genotypes to allow for accurate quantitative analysis. Each strain was imaged on at least three separate days, and the resulting data sets were combined for analysis. Image quantification was carried out using ImageJ (v1.48, NIH) with Fiji plugins. In short, VNC regions were cropped into rectangular regions (30 µm wide × 2.5 µm high) encompassing only the nerve cord and were projected as a sum in the Z direction. Mean signal intensity was projected along the *x*-axis (perpendicular to the cord), and fluorescence intensity was quantified by calculating the area under the curve, excluding background signal.

#### Fly stocks and maintenance

Fly stocks were kept on standard cornmeal-molasses food. Control w1118 (w-) was obtained from the Bloomington Drosophila Stock Center (#6326). dCreld mutants were generated by homologous recombination-mediated gene targeting.^19^

### Investigation of locomotion in *Drosophila melanogaster* CRELD mutants

#### Analysis of larval locomotion

Ten staged L1 larvae from egg collections on apple juice plates with fresh yeast paste of control (w1118) and dCreld (CreldΔ51) were transferred to fresh plates and grown at 25°C for 3 days. Five late third instar larvae were transferred to PBS plates. Crawling was monitored for 1 min. Pictures were taken every second using the Infinite shot app. Pictures were analyzed with the Manual Tracking Plugin in ImageJ and the total distance and mean speed of each larva were measured.

#### Analysis of adult negative geotaxis

The rapid iterative negative geotaxis assay was used to measure adult locomotion.^25^ Fifteen 5-day old adult female flies of each genotype were separated into fresh vials and were allowed to recover from anesthesia for at least 24 h. Flies were placed in glass vials in a scaffold that allows the simultaneous analysis of several genotypes. The scaffold was tapped on the ground, which induces negative geotaxis behaviour in healthy flies. Fly locomotion was filmed, and frames from 3, 6 and 20 s were analyzed. The distance from the 10 cm threshold to fly head was measured for each individual, and the climbing distance was calculated.

### Statistical analysis

Statistical analyses were performed using Prism software (GraphPad). For comparisons between two groups, the nonparametric Mann–Whitney test was used to compare fluorescence intensity between +/+ and C187Y/C187Y. For Drosophila locomotion analysis, an unpaired, two-tailed Student’s t-test assuming heteroscedasticity was applied. For comparisons involving more than two groups, the nonparametric Kruskal–Wallis test or Fisher’s exact test with Bonferroni correction was used, as indicated in the corresponding figure legends. For all tests, ****P < 0.0001; ***P < 0.001; **P < 0.01; *P < 0.05.

## Results

### Clinical case

**Figure 1:**
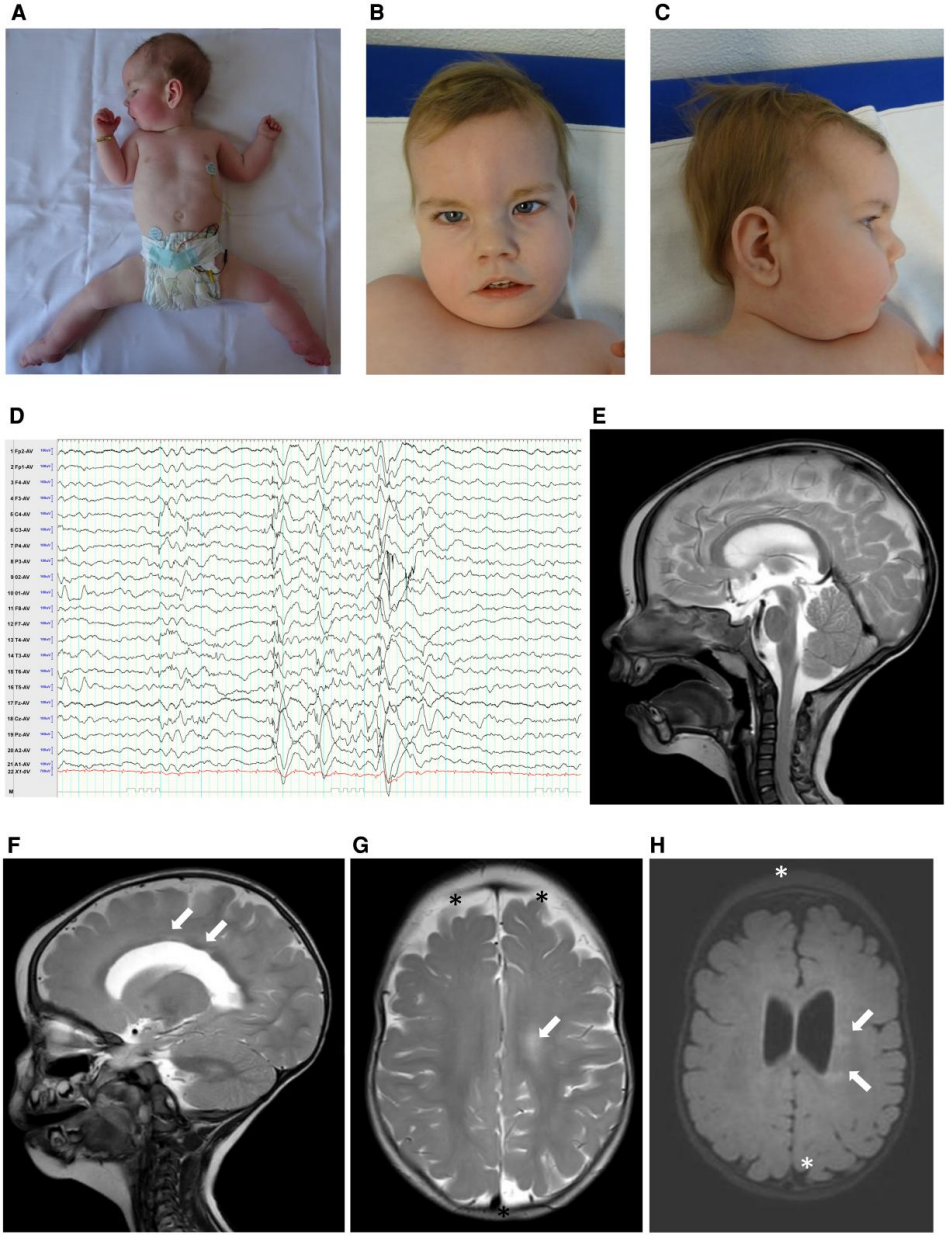
**Clinical presentation electroencephalogram and brain MRI.** The index patient suffering from severe muscle weakness with limited anti-gravity movements at the age of 8 months (**A**). Facial stigmata included a prominent forehead, deep-set ears, hypertelorism, broad nose, epicanthus and retrognathia at the age of 14 months (**B, C**). EEG at 9 months showing the voltage on the *Y*-axis (each blue bar indicates the scale in microvolts) and the time on the *X*-axis (the interval between two solid vertical lines corresponds to 1 s). The EEG showed spike-wave complexes singular and in small groups located centroparietal on both sides, but predominantly on the left side. Occasional spread to the temporal (T5) was noted. Generalized spike-slow wave complexes correlated with myoclonic seizures (**D**). Cranial MRI at 12 months of age showed no major brain malformations (**E**). Unspecific periventricular hyperintensities (arrows) were noted on T2-weighted (**F, G**) and on fluid-attenuated inversion recovery (FLAIR) images (**H**), as well as an enlargement of the outer cerebrospinal fluid space (asterisk; **G, H**).

The male patient was born as the first child of non-related healthy German parents. Prenatally reduced fetal movements, and polyhydramnios were noted. After an uneventful delivery at 39 + 3 weeks of gestation (APGAR 7/8/8, pH: 7.37; birth weight 2900 g [P17], body length 51 cm [P41], head circumference 33 cm [P3]), he presented as floppy infant requiring respiratory support via continuous positive airway pressure in the first five days of life and feeding via nasogastric tube. Clinical examination revealed arthrogryposis with pes equinovarus of both feet, mild contractures of the elbows and camptodactyly of the right hand (Fig. 1A). In addition, the boy presented with cryptorchidism, pectus excavatum and arched palate. Facial stigmata included a prominent forehead, deep-set ears, hypertelorism, broad nose, epicanthus and retrognathia (Fig. 1B and C). Cranial and abdominal ultrasounds were unremarkable; the echocardiogram showed a small patent foramen ovale without hemodynamic relevance.

Follow-up of the boy showed non-progressive proximal and axial pronounced muscle weakness and developmental delay, including weak cry, obstipation and malnutrition because of poor sucking. Creatine kinase (CK) activity was not elevated (max. 154 U/l at 14 months). Growth and weight gain improved after administration of high-calorie food. Because of his rigid club feet (Pirani Score 5.5) cast therapy and achillotomy were performed. From 5 months on, the patient developed focal seizures presenting with myoclonus of the upper extremities and face. Electroencephalogram (EEG) at 7 and 8 months showed alternating, but predominantly left-sided centroparietal sharp waves, singular and in small clusters (Fig. 1D).

In addition, generalized sharp-wave complexes were noted as a correlate of myoclonic seizures. At 7 months of age, he showed some antigravity movements with the upper extremities but not the lower extremities and showed no head control. Ophthalmologic examination revealed a convergent strabismus. At 8 months of age, the patient presented with an epileptic status with predominantly myoclonic seizures and despite anti-seizures medication, he had several short focal myoclonic seizures per day with secondary generalization two to three times per week during the following month. Levetiracetam, ethosuximide and lacosamide did not significantly reduce the seizures, but less epileptiform activity was noted on the EEG at 9 months. After changing the anticonvulsive therapy to levetiracetam, valproate, and clobazam, the seizure frequency decreased. In the EEG at 11 months, only occasionally centroparietal alternating but mostly left-sided spike wave complexes were noted. At 11 months of age, the boy was able to move his hands to his mouth and grab his feet with his hands to move them to his mouth. However, he was still unable to bring his feet together. In a prone position, he was able to lift his head for a moment. His movements were faster and more variable. Clinically, he was attentive, he smiled and seemed to recognize his parents.

Electromyography at 14 months of age showed a myopathic pattern without a significant decrement. We observed clonus after eliciting the patellar tendon reflex during the examination. At 15 months of age, the boy had prolonged status epilepticus induced by a febrile infection, which was terminated only by deep sedation. Extubation was successful after four days, but the patient presented increased muscle weakness, requiring a percutaneous endoscopic gastrostomy tube for feeding. Valproate dosage was further increased to reach therapeutic blood levels. This resulted in a significant reduction in seizures. Additionally, after informed consent, a therapy trial with pyridostigmine, an inhibitor of cholinesterase, was initiated. At the last follow-up at 17 months (body weight 9.3 kg [P7], body length 83 cm [P63], OFC 47 cm [P14]) the boy presented severe weakness with the ability of slow antigravity movements of the lower limb but was not able to turn himself, sit or hold his head. Apart from residual club feet, no contractures were present. Motor ability was assessed via CHOP INTEND (Children’s Hospital of Philadelphia Infant Test of Neuromuscular Disorders) and resulted in 34/64 points. At 23 months of age, he had a cardiorespiratory arrest during a prolonged status epilepticus and after initial successful reanimation, followed by a medical/ethical counselling of the parents, the boy died under palliative treatment.

In the search for the underlying etiology, a cranial magnetic resonance imaging (MRI) was performed at 7 and 12 months of age and showed non-progressive periventricular hyperintensities on T2-weighted images and an enlargement of the outer cerebrospinal fluid space (Fig. 1E–H). Magnetic resonance of the brain at 12 months did not unravel any pathological metabolites.

Muscle biopsy was performed at 1 year of age from the left quadriceps femoris and showed increased fibre size variability with hypertrophic fibres 15–30 µm, up to 45 µm and atrophic fibres of 5–8 µm in diameter. The endomysial connective tissue was mildly increased and there was no necrosis or centralized nuclei. ATPase staining revealed a predominance of type 1 fibres (data not shown) without grouping. A few neonatal myosin-positive fibres were present as sign of regeneration. A small motor nerve was included in the muscle biopsy and showed regular density and size of myelinated small axons. Overall, the muscle biopsy was pathogenic and interpreted as neurogenic.

The mandatory German newborn screening for metabolic diseases (www.screening-dgns.de) was unremarkable. Extensive metabolic testing revealed only unspecific changes. Low cerebrospinal fluid concentrations of most amino acids, including alanine and methionine, were reported. In addition, a low concentration of homovanillinacid with normal 5-hydroxyindoleacetic acid was suggesting a dopaminergic transmission disorder, but a therapy trial with L-Dopamin/Carvidopa did not show any effect. The karyotype was 46, XY and Array-CGH were unremarkable. Fluorescence in situ hybridization analysis of the 15q11.2 region did not reveal any structural alterations as a possible cause of Prader–Willi or Angelman syndrome, respectively.

### Identification of compound heterozygous variants in *CRELD1*

The WES was performed in order to investigate the etiology of the disease, and the resulting variants were filtered according to various criteria (Supplementary Methods and Supplementary Table 3). No likely pathogenic variants were identified in genes known to be associated with neuromuscular diseases, suggesting that the phenotype was caused by variants in a novel gene.

**Figure 2:**
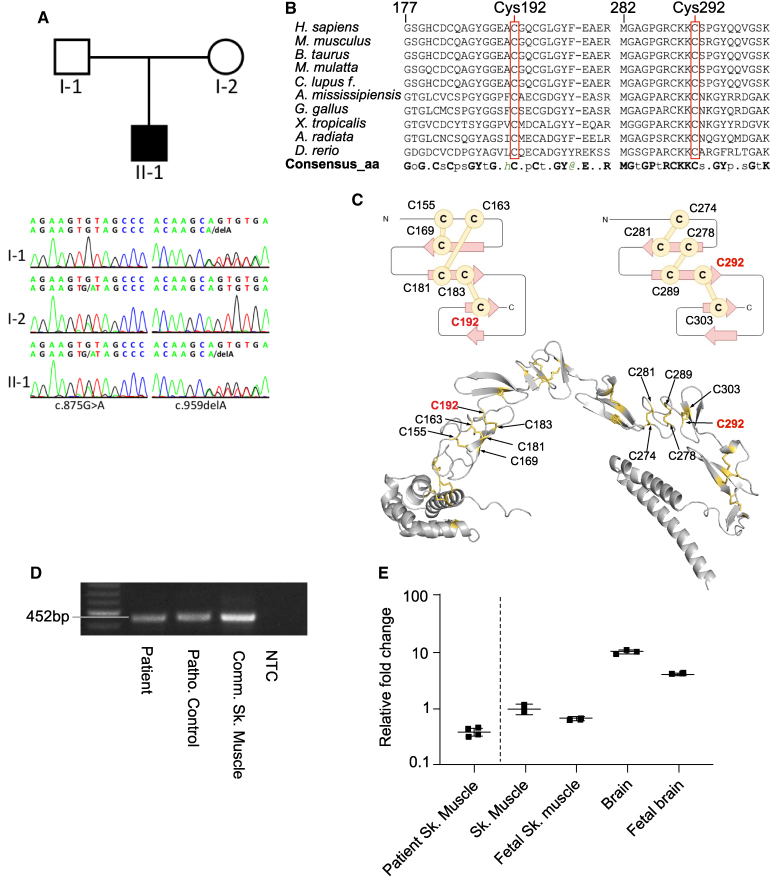
***CRELD1* missense variants and expression analysis.** Pedigree of the family, illustrating the non-consanguineous parents and the index patient (II/1). Compound heterozygous inheritance was confirmed by Sanger sequencing (**A**). The amino acid exchange of the missense variant detected in our patient (p. Cys292Tyr) and the missense variant reported in ClinVar (p. Cys192Tyr) affect evolutionarily highly conserved residues. The alignment was performed with Promals3D, consensus_aa = consensus amino acids; more details can be found in Supplementary Methods. (**B**). Diagram displaying that both cysteines (p. Cys192 and 292) are located in the EGF-like domains and form disulphide bonds that are essential for the domain structure; the CRELD1 EGF-like domains and interchain disulphide bonds were modelled with AlphaFold (https://alphafold.ebi.ac.uk) and visualized with PyMOL. Predicted disulphide bonds are shown in sticks and highlighted in yellow (**C**). RT-qPCR analysis of total RNA from the patient’s skeletal muscle sample, and from commercial skeletal muscle samples. NTC = no template control. No abnormal splicing was detected on the agarose gel. The full-size uncropped gel is in Supplementary material (**D**). RT-qPCR results showing downregulation of *CRELD1* expression in the patient skeletal muscle compared to the commercial skeletal muscle as well as the variable *CRELD1* expression in the brain, fetal brain and skeletal muscle tissues (**E**). Each data point represents the average of three technical replicates. For the patient sample, *N* = 4; for the other samples, *N* = 3. Sk. = skeletal.

Further expanded filtering revealed two heterozygous variants in *CRELD1*. These variants were confirmed by Sanger sequencing and shown to segregate in a compound heterozygous pattern in the parents, with one allele inherited from each parent (Fig. 2A).

The first variant was a novel missense variant in exon 8 (c.875G>A, p.Cys292Tyr). It had not been reported in any database and was predicted to be deleterious by several online in silico tools (SIFT, PolyPhen and CADD). The second variant was a frameshift variant in exon 9 (c.959delA, p.Gln320Argfs*25). It was reported as a rare variant in population databases (rs759473511, gnomAD allele count 84/282702, allele frequency: 0.000297) and once in our in-house database. A recent study reported five patients with this deletion (p. Gln320Argfs*25) in a compound heterozygous state with the CRELD1 missense variant (p. Cys192Tyr).^20^ All these patients matched the phenotype of our patient, presenting congenital muscle weakness, global developmental delay and focal epilepsy. Interestingly, both the p. Cys292Tyr found in our patient and the p. Cys192Tyr variants affect evolutionarily highly conserved cysteine residues, involved in disulphide bonds that are important for stabilizing the domains in which they are located (Fig. 2B and C). Interestingly, functional studies have reported that mutations in conserved cysteine residues can partially impair CRELD1 function.^18^

### Characterization of *CRELD1* patient expression

We performed RT-PCR on the patient’s muscle sample to detect possible effects on splicing caused by the c.875G>A, p. Cys292Tyr variant; however, we could not detect an abnormal fragment as a sign of splicing alterations on the agarose gel (Fig. 2D). Sequencing of the fragment spanning exons 5 to 9 also revealed no splicing disruption and heterozygous expression of the missense variant in exon 8, with the truncating variant located more c-terminal in exon 9.

RT-qPCR was performed to show the expression changes associated with the c.959delA, p. Gln320Argfs*25 variants. The *CRELD1* expression in the patient muscle was reduced more than 50% compared to the commercial RNA from skeletal muscle controls (Fig. 2E). In addition, we performed RT-qPCR on fetal muscle and adult brain control tissues and showed high expression of *CRELD1* in both brain and developing muscle in agreement with Rupp *et al*.^13^ (Fig. 2E).

### CRELD1 domain structure and homologues in *C. elegans* and *D. melanogaster*

**Figure 3:**
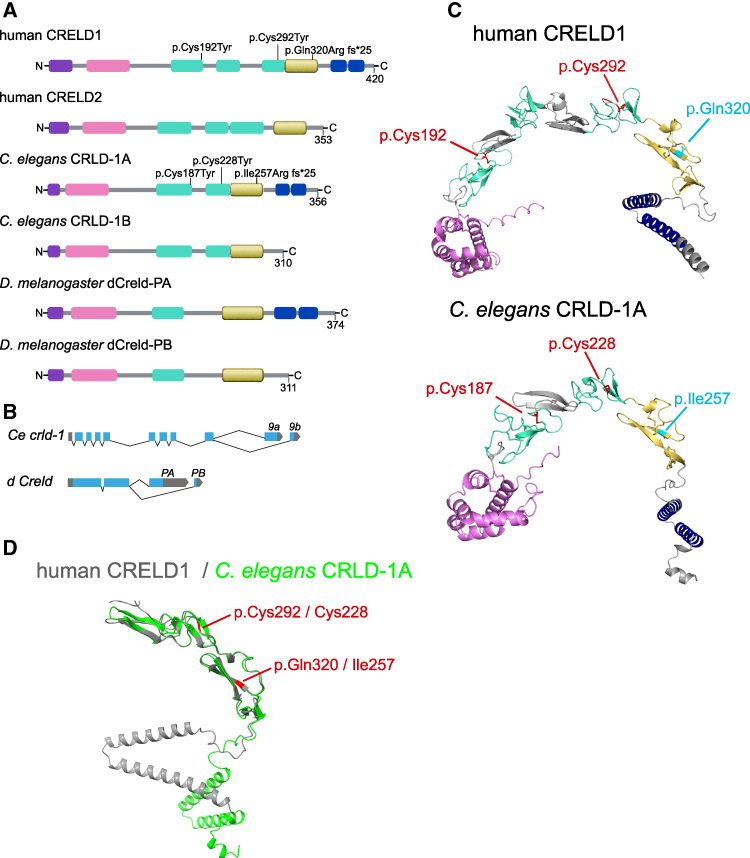
**Domain architecture of the human, *C. elegans* and *D. melanogaster* CRELD proteins.** Human CRELD1 (NP_001070883.2) consists of an N-terminal signal peptide (violet), the WE domain (pink) specific for the CRELD protein family, two EGF-like domains (green) followed by an EGF-like-cbEGF tandem domain consisting of an EGF-like and a Ca2+-binding EGF-like (cbEGF) domain (yellow) and two C-terminal transmembrane domains (blue) (**A**). The human *CRELD2* gene encodes a homologous protein lacking only the transmembrane domains (**A**). *C. elegans* and *D. melanogaster* have only one *CRELD* gene, but express two different alternatively spliced isoforms, one with and one without the transmembrane domains (**A, B**). The alternative splicing of the last exons in *C. elegans* (exon *9a* or *9b*) and *Drosophila* (exon *PA* or *PB*) gives rise to the two different CRELD1 isoforms (**B**). The domain architecture is predicted to be similar to human CRELD1, which contains an additional EGF-like domain (**A**). Modelling of the protein structure with AlphaFold and visualization with Pymol of human CRELD1 and *C. elegans* CRLD-1A highlight the similar tertiary structure. Cysteines and disulphide bonds are shown in sticks. The cysteine mutated in our patient forms a disulphide bond in the third EGF-like domain. Human Cys 192 and Cys 292 and *C. elegans* Cys 187 and Cys 228 are highlighted in red. Human Gln 320 and *C. elegans* Ile 257 are highlighted in cyan (**C**). AlphaFold predicted structures alignment using ChimeraX16.1. The C-terminal domains encompassing the cbEGF and the two transmembrane domains from both human CRELD1 and *C. elegans* CRLD-1A were aligned. Human CRELD1 (from Asp 247 to Arg 420 residue) is shown in grey, *C. elegans* CRLD-1A (from Ile 192 to Tyr 356) is in green. Human Cys 292 and Gln 320 and *C. elegans* Cys 228 and Ile 257 are highlighted in red (**D**).

*CRELD1* is a highly evolutionarily conserved gene. Vertebrates also have the gene *CRELD2*, which is homologous to *CRELD1* except for the absence of the transmembrane domain. Invertebrates such as *Drosophila melanogaster* or *C. elegans* express two different isoforms generated from one gene by alternative splicing, homologous to the human *CRELD1* and *CRELD2*, respectively (Fig. 3A and B).

CRELD1 is an endoplasmic reticulum-localized transmembrane protein with the functional domains facing the ER lumen.^13,17,18^ The protein has an N-terminal signal peptide for ER import and two C-terminal tandem transmembrane domains (Fig. 3A). It contains two types of functional domains. First, the so-called WE domain is a unique N-terminal region rich in glutamic acid and tryptophan residues.^13^ This WE domain has been shown to contain a CXXC motif that supports PDI activity.^18^ The presence of CXXC motifs in mammalian CRELD1 suggests that this PDI function, which is essential for the correct formation of disulphide bonds in client proteins, has been conserved throughout evolution. In addition, the CXXC motifs of human CRELD2 have been demonstrated to have disulphide isomerase activity.^26^

Second, CRELD1 possesses a cysteine-rich central region composed of multiple epidermal growth factor-like (EGF-like) domains. These domains are characterized by six conserved cysteine residues that conventionally form disulphide bonds in a I–III, II–IV and V–VI pattern. Structurally, they predominantly consist of a double-stranded β-sheet, which is followed by a shorter β-sheet near the C-terminus.^27^ Calcium-binding EGF-like (cbEGF-like) domains contain a calcium-binding site.^27^ EGF-like domains are important for protein-protein interactions as participants in signalling pathways and have structural functions, acting as sensors to control protein rigidity or as spacers to position connected domains within reach of an interaction site.^27^

The exact arrangement of the EGF domains in human CRELD remains uncertain in the absence of a crystal structure. The SMART algorithm (http://smart.embl-heidelberg.de/) predicts five models as described by Rupp *et al*.^13^ However, the model with the highest probability scores predicts two EGF-like domains followed by an EGF-like-cbEGF tandem domain (Fig. 3A), which is consistent with the in-silico prediction of AlphaFold^28^ (Fig. 3C). *C. elegans* and *Drosophila* share a similar CRELD1 protein domain structure, but lack an EGF- and a cbEGF-like domain compared to human CRELD1 (Fig. 3A).

### Generation and characterization of a *CRELD1* knock-in *C. elegans* model using CRISPR/Cas9

We took advantage of the conservation of CRELD1 between *C. elegans* and humans to test the impact of the compound variant identified in our patient (p. Cys292Tyr/p. Gln320Argfs*25). We modelled this variant in *C. elegans* using CRISPR/Cas9 technology. Since CRLD-1A is required for the biogenesis of *C. elegans* heteromeric levamisole-sensitive AChRs (L-AChRs),^18^ which are similar to vertebrate AChRs at mammalian NMJs, we analyzed AChR expression as a proxy for CRELD function. *Crld-1a* knockout animals show a substantial decrease in L-AChRs at the NMJ resulting in reduced sensitivity to the nematode-specific cholinergic agonist levamisole. They are completely paralyzed at high concentrations of levamisole within a few hours, but subsequently adapt within 12–16 h and recover motility in contrast to the wild type.^18,29^

**Figure 4:**
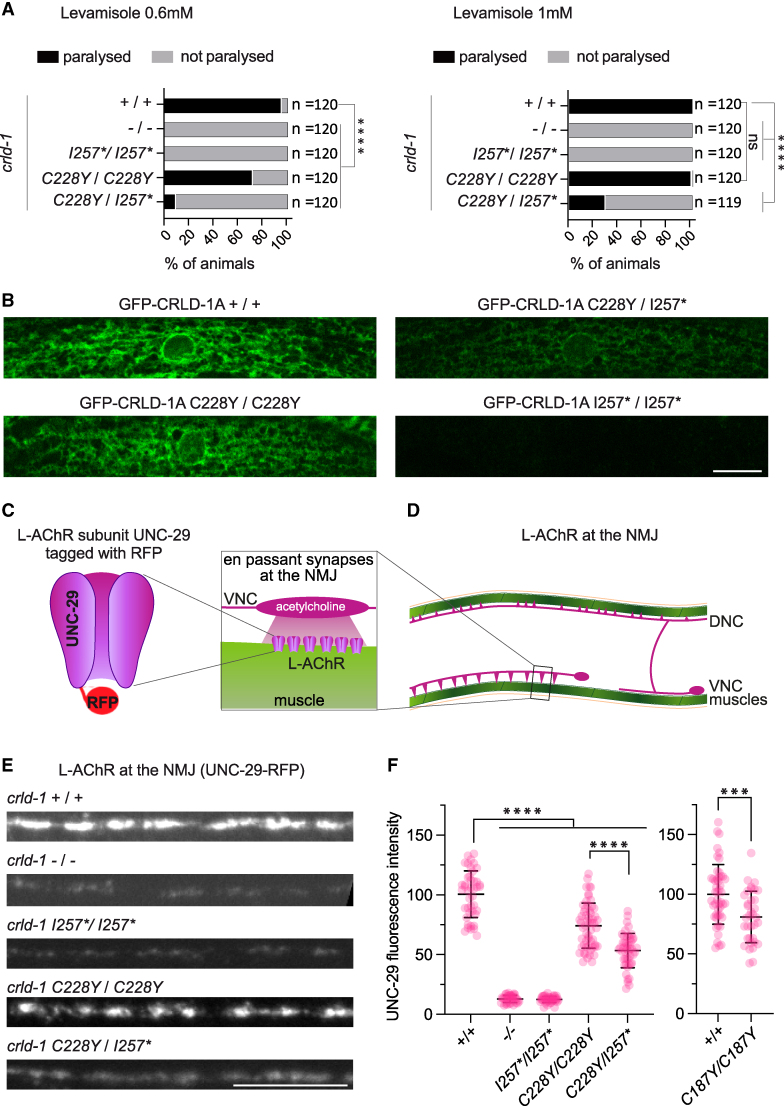
**The patient’s compound heterozygous variant affects L-AChR expression at the NMJ.** Levamisole test on both wild types (indicated as +/+), *crld-1* knock outs (indicated as −/−), *crld-1* Cys228Tyr and Ile257RFs*25 knock-ins (indicated as *C228Y/C228Y* and I257*/I257*, respectively) and *crld-1* compound heterozygous mutants (indicated as C228Y/I257*). C228Y/I257* mutants are largely resistant to levamisole. Experiments were repeated six times. ns = not significant *P* = 0.4979 for *C228Y/C228Y on* 1 mM levamisole, ****P < 0.0001, after Bonferroni correction, Fisher’s exact probability test. Grey bars indicate the percentage of moving animals after overnight exposure to either 1 mM or 0.6 mM levamisole, and black bars indicate the percentage of paralyzed animals, the number of animals tested for each genotype is indicated (**A**). Distribution of GFP-CRLD-1A in muscle cells of *gfp-crld-1a* knock-in animals carrying also the different human *CRELD1* mutations found in the patient. In compound heterozygous mutants (C228Y/I257*), the GFP-CRLD-1a pattern is much less intense as compared to the control (+/+). Scale bars = 10 μm (**B**). Schematic representation of L-AChR in which the subunit UNC-29 is tagged with RFP (**C**). Organization of excitatory cholinergic NMJs in *C. elegans*. Muscle cells (green) are innervated by cholinergic motoneurons (purple). L-AChR are clustered in front of cholinergic boutons (inset) along the VNC and DNC.^34^ (**D**). Confocal imaging of the L-AChR reporter UNC-29::tagRFP at the ventral nerve cords of control (+/+), crld-1(I257*) and *crld-1(C228Y)* alone or crossed with crld-1(I257*) mutant worms (C228Y/I257*). Scale bars = 10 μm (**E**). Quantification of UNC-29::tagRFP fluorescence at the ventral nerve cords. Each dot represents the fluorescence intensity of UNC-29::tagRFP measured at the VNC of a worm. Data are normalized on control mean and presented as mean ± SD; control, +/+: *n* = 43 animals, *crld-1(−/−): n* = 37 animals, crld-1(I257*/I257*): n = 40 animals, *crld-1(C228Y/C228Y): n* = 57 animals, *crld-1(C228Y)* crossed with crld-1(I257*), C228Y/I257*: *n* = 41; experiments were repeated four times. ****P < 0.0001, Kruskal–Wallis test. Mann–Whitney test was applied to compare *C187Y/C187Y* animals (*n* = 35 animals) with their control (*n* = 48 animals). ***P < 0.001 (**F**).

To determine whether a missense variant affecting a conserved cysteine has deleterious effects, we introduced the same missense variant as in the patient at the orthologous positions in the *C. elegans crld-1a* gene. Based on the sequence alignment and AlphaFold prediction in *C. elegans* Cys228 corresponds to human Cys292 (Fig. 3A and C). We generated three independent knock-in lines expressing this missense variant (*crld-1a(C228Y)*) and tested their sensitivity to levamisole. C*rld-1a(C228Y)* animals were not resistant to 1 mM and weakly resistant to 0.6 mM levamisole (Fig. 4A and Supplementary Fig. 1A), suggesting that this missense cysteine-to-tyrosine variant partially impairs CRLD-1 function.

In the Gln320Argfs*25 variant, the mutation of Gln320 to an Arg is followed by a frameshift that introduces 25 unrelated amino acids ending with a premature stop in the first beta sheet of the cbEGF domain. The AlphaFold-predicted structure of the C-terminal region of human CRELD1 was aligned with that of *C. elegans* CRLD-1 using ChimeraX. This region encompasses the cbEGF and the transmembrane domains. We found that based on structure alignment human Gln320 corresponds to *C. elegans* Ile257 (Fig. 3D). Using CRISPR/Cas9, we replaced this residue with an Arg, followed by the same 25 non-native C-terminal amino acid residues found in the patient (Ile257Argfs*25 mutation), in order to generate the truncated CRLD-1 expressed in the patient carrying the Gln320Argfs*25* variant. We generated three independent knock-in lines (I257*) that were tested on levamisole. We found that crld-1 (I257*) mutants exhibited resistance to both 0.6 and 1 mM levamisole, a phenotype similar to that observed in *crld-1(−)* null mutants (Fig. 4A and Supplementary Fig. 1B). To model the compound variant found in the patient, we crossed the Cys228Tyr mutant with I257Rfs*25 mutant. Animals containing the compound variant (C228Y/I257*) were largely resistant to levamisole (71% and 91% of animals were resistant to 1 and 0.6 mM levamisole, respectively), as shown in Fig. 4A. Interestingly, the I257Rfs*25 variant showed antimorphic activity, as compound heterozygous animals expressing the *crld-1(−)* null mutation in combination with *crld-1(C228Y)* were much less resistant to levamisole than C228Y/I257* (Supplementary Fig. 1C).

Since CRLD-1A is localized in the ER in muscle cells, where it promotes the assembly of AChR subunits,^18^ we decided to look at the expression pattern of GFP-CRLD-1A in muscle cells carrying the patient variants. In homozygous c*rld-1a(C228Y)* and in the compound heterozygous mutants (C228Y/I257*), the typical ER pattern was present, but the GFP-CRLD-1 network and its perinuclear localization were less pronounced and well-delineated, particularly in the compound mutant. On the contrary, in the homozygous I257* mutant, CRELD-1 expression was lost (Fig. 4B). To test if the truncated I257* CRLD1 may be expressed and secreted, we looked at coelomocytes. Coelomocytes in *C. elegans* are scavenger cells that continuously and nonspecifically endocytose fluid from the pseudocoelomic cavity. Fluorescently-tagged proteins secreted into the pseudocoelom from body wall muscle cells are endocytosed and degraded by coelomocytes. We used DIC to identify the coelomocytes and observed GFP-CRLD-1 signal in the coelomocytes of I257* animals (Supplementary Fig. 2).

To analyze L-AChR distribution, we then used a knock-in strain carrying the *unc-29(kr208::rfp)* allele, in which the red fluorescent protein TagRFP is inserted at the endogenous locus of the UNC-29 receptor subunit (Fig. 4C). In these animals, L-AChRs form visible clusters at NMJs along both the ventral and dorsal nerve cords (DNC) (Fig. 4D and E). We found that the fluorescence intensity of L-AChRs present at the ventral nerve cord was decreased by 45% in animals expressing the compound heterozygous variants (C228Y/I257*) as compared to the wild type (Fig. 4F). We also observed a 25% decrease in fluorescence in *crld-1a(C228Y)* animals (Fig. 4F). Taken together, our data strongly suggest that the compound heterozygous variant found in the patient is pathogenic and impairs CRELD1 function.

Finally, we tested the effect on AChRs of the recently described missense variant p.Cys192Tyr, which leads to phenotypes similar to our patient when present either in *trans* with the p. Gln320Argfs*25 or in homozygous configuration.^20^ According to sequence alignment and AlphaFold prediction, human Cys192 corresponds to Cys187 in *C. elegans* (Fig. 3A and C). We generated three independent knock-in lines expressing this variant (*crld-1a(C187Y))*, which were tested for their effect on levamisole sensitivity and L-AChRs levels at the NMJ. Both assays showed that this variant (C187Y) induced phenotypes similar to those observed for the missense variant in our patient (C228Y): homozygous *C187Y* animals were paralyzed at 1 mM levamisole and weakly resistant to 0.6 mM levamisole, and caused a 15% decrease in the AChR fluorescence intensity at the NMJ (Supplementary Fig. 1A and Fig. 4F).

### CRELD is required for locomotion in both *C. elegans* and *Drosophila* models

**Figure 5:**
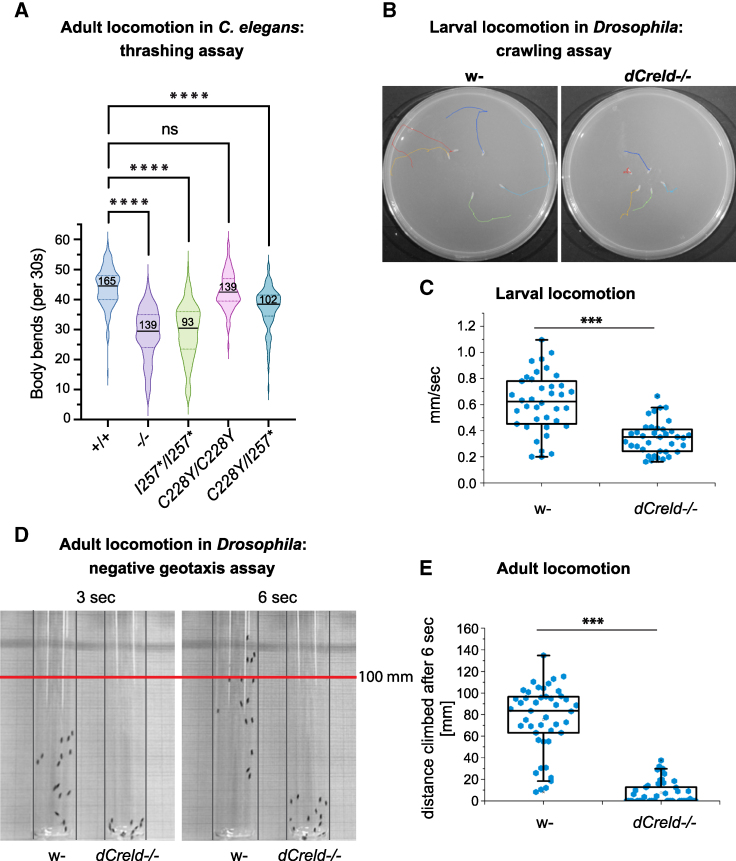
**CRELD is required for locomotion in both *C. elegans* and *Drosophila* models.** Thrashing assay for *C. elegans* locomotion: body bends frequency at day 1 of adulthood of wild types (indicated as +/+), *crld-1* knockouts (indicated as −/−), *crld-1* Cys228Tyr and Ile257RFs*25 knock-ins (indicated as *C228Y/C228Y* and I257*/I257*, respectively) and *crld-1* compound heterozygous mutants (indicated as C228Y/I257*). Black line: median value, dotted lines: lower and upper quartiles, *n*: number of animals scored in at least three independent experiments; ns = not significant, ****P < 0.0001, Kruskal–Wallis test (**A**). Crawling assay for *Drosophila* larval locomotion: single larvae are semi-automatically tracked (green line represents distance crawled in 20 s) (**B**). Speed in mm/s. Dots represent individual larvae, w-*n* = 39, *dCreld−/− n* = 37 (**C**). Negative geotaxis assay for adult locomotion: flies are placed in glass tubes and tapped on the ground. Climbing is recorded (**D**). Distance from the fly head to the bottom after 6 s. Boxes in box plots represent the interquartile range and median; whiskers represent the minimum and maximum. Significance tested by unpaired, two-tailed Student’s t-test assuming heteroscedasticity (*** indicates *P* < 0.001). Dots represent individual animals, w-*n* = 45, *dCreld−/− n* = 45 (**E**). In panels ***C*** and ***E***, each experiment was performed in at least three independent biological replicates.

Previous data indicate that the absence of CRELD1 may impact *C. elegans* locomotion, though this has not been extensively characterized.^18^ To investigate this phenotype in greater depth, particularly in the context of patient-derived *CRELD1* variants, we performed thrashing assays to quantitatively assess locomotor behaviour. We observed a significant reduction in the body bend frequency in *crld-1* null mutants, as well as in animals carrying either the I257* variant or the compound C228Y/I257* variants, relative to wild-type controls (Fig. 5A). In contrast, animals expressing only the C228Y variant exhibited mobility comparable to wild type (Fig. 5A).

To further assess the functional conservation of *CRELD1* in regulating locomotion, we extended our analysis to *Drosophila* models. dCreld has been implicated in the ER-mitochondrial transfer of phospholipids with effects on mitochondrial respiratory complex I activity. In *Creld* mutants, dopaminergic neurons are inactive due to low production of hydrogen peroxide at complex I, leading to a marked locomotor deficit in adult flies.^19^ Here, we examined larval and adult locomotion in wild type and *dCreld* mutants (Fig. 5B–D). In the crawling assay for larval locomotion, mutants showed a significantly reduced distance travelled in a given time compared to wild type (Fig. 5B and C). Second, we performed a negative geotaxis assay for adult locomotion. Flies were placed in glass tubes and tapped on the floor. Mutants were unable to climb up the tube against gravity, whereas wild-type animals easily climbed up the glass wall (Fig. 5D and E). We analyzed *Drosophila* larvae NMJs and found that their morphology was unchanged in homozygous *dCreld* mutants (Supplementary Fig. 3B, compare with heterozygous control in Supplementary Fig. 3A). Although *Drosophila* NMJs are glutamatergic and not cholinergic, we tested for a role of dCreld in AChRs biosynthesis by ectopically expressing GFP-tagged nAChRs. This revealed reduced fluorescence intensity in *dCreld* homozygous mutant NMJs compared to heterozygous controls, suggesting that dCreld is also required for AChR maturation in flies (Supplementary Fig. 3B, compare with heterozygous control in Supplementary Fig. 3A). Altogether, the *Drosophila* and *C. elegans* data support a conserved role for CRELD1 in the regulation of locomotion across evolution.

## Discussion

This work identified a *CRELD1* compound heterozygous pathogenic variant as responsible for a rare disease characterized by arthrogryposis, muscle weakness, severe global developmental delay and epilepsy. Mutation modelling in *C. elegans* confirmed the pathogenicity of these variants and further provided a tentative mechanism for the physiopathology of this disease.

CRELD1 is a multimodular protein. The compound heterozygous *CRELD1* variants identified in our patient introduce a missense variant p.Cys292Tyr on one allele. This cysteine, located in the first cbEGF-like domain, is involved in the formation of a conserved disulphide bridge with Cys303, according to AlphaFold CRELD1 protein structure prediction (Fig. 2C). The *C. elegans* point mutation Cys228Tyr, corresponding to the human Cys292 position, mildly affects both CRLD-1 and AChR expression when homozygous. On the other allele, the patient has a frameshift p.Gln320Arg with the deletion of the last 100 amino acids. These residues encompass the cbEGF and two C-terminal transmembrane domains. The resulting truncated protein is likely to be secreted, leading to a complete loss-of-function, as the localization to the ER membrane has been shown to be essential.^16,18^ Modelling this deletion in *C. elegans* confirmed this hypothesis as Ile257Argfs*25 mutants behave like null mutants and CRLD-1 is no longer present in muscle cells.

The compound heterozygous configuration (C228Y/I257*) causes a severe phenotype according to mobility assay, levamisole test and quantification of AChR levels at the NMJ in *C. elegans*. The reticular pattern of GFP-CRLD-1A could still be seen, although it was much weaker than the wild-type CRLD-1 pattern. The truncated version of CRLD-1, in conjunction with the hypomorphic C228Y CRLD-1 point mutation, likely has a significant adverse effect on the protein secretory pathway, potentially leading to the formation of non-functional or misassembled AChRs. Interestingly, the levamisole sensitivity of the *C228Y* point mutant over a *crld-1(−)* null mutant is similar to the wild type (Supplementary Fig. 1C), supporting our hypothesis that the I257* mutation might ‘poison’ AChR receptor maturation in the context of the C228Y variant.

Based on mouse genetic data, a complete biallelic *CRELD1* loss-of-function in humans is probably not viable. Monoallelic heterozygous variants might reduce expression and contribute in a multi-causal manner to the susceptibility to heart defects, especially in combination with chromosomal aberrations such as Down syndrome.^25^ Based on our data, a truncating variant with antimorphic activity combined with a missense variant affecting one of the functional domains of CRELD1 is able to cause severe multisystem disease as in our patient, including muscle weakness, arthrogryposis, developmental delay and epilepsy. Such combination likely defines the rare neurodevelopmental genetic disorder associated with CRELD1 dysfunction identified in our patient.

Recently, biallelic variants in *CRELD1* were identified in 18 individuals from 14 families.^20^ The affected individuals had a range of symptoms similar to those observed in our patient, including developmental delay, early-onset refractory epilepsy and hypotonia. Most affected individuals carried a frameshift mutation in *trans* with a missense allele, with one recurrent variant, p.(Cys192Tyr), found in 10 families. To characterize the effect of these CRELD1 variants at first, the authors showed that knockdown of *creld1* in *X tropicalis* tadpoles resulted in developmental defects and increased susceptibility to induced seizures compared to controls. They subsequently overexpressed the CRELD1 missense patients’ variants in *X. tropicalis* embryos to assess their effect on CRELD1 function. They found that ectopic overexpression of wild-type human CRELD1 induced defects in craniofacial development in *X. tropicalis*, whereas the patient missense variants had a reduced ability to induce these defects, suggesting that the CRELD1 missense variants are less functional than the reference protein. However, this study did not investigate the precise effect of each variant on the CRELD1 protein. In particular, the frameshift mutations were not characterized and assumed to be equivalent to a null allele. In addition, this strategy could not directly test the impact of biallelic variants. *C. elegans* is one of the few model organisms that allows a rapid and targeted analysis of such biallelic variants. In the case of our patient, we were able to show that the frameshift mutation Ile257Argfs*25 negatively impacts CRELD1 function when present in *trans* with the missense variant Cys228Tyr. In contrast, the null allele of *crld-1* in *trans* with *C228Y* behaves almost like the wild type. Our findings provide strong evidence that this specific type of compound variant is responsible for a multisyndromic disease state of the patient.

CRELD1 has been shown to function as both a chaperone and a PDI and is required for the biosynthesis of several substrates. However, the dependence of AChRs on CRELD1 provides an interesting hypothesis to explain the clinical features observed in our patient. Mutations in several AChR subunit genes have been associated with genetic forms of arthrogryposis (*CHRNA1*, *CHRNB1*, *CHRND*, *CHRNE*, *CHRNG*).^30^ AMC is a condition recognized in the neonatal period by the presence of multiple congenital contractures associated with muscle weakness throughout the body.^31^ AMC is characterized by primary skeletal muscle involvement followed by brain involvement, with the most common associated neurological symptoms being epilepsy, intellectual disability and brain malformations.^6^ Our patient presented with fetal hypokinesia and arthrogryposis. At follow-up, the child showed severe muscle weakness, developmental delay, severe epilepsy and cognitive disability, representing the spectrum of syndromes associated with arthrogryposis. Since CRELD1 controls the maturation of muscle AChRs, we could speculate that the compound *CRELD1* variants found in the patient could reduce the amount of mature AChRs reaching the muscle surface, thus accounting for an AMC-like phenotype. Indeed, we have previously demonstrated that a biallelic variant of UNC-50, which encodes a protein required for AChR biogenesis, is responsible for arthrogryposis through a likely loss of AChRs at the NMJ.^12,32^

AChR dysfunction is also associated with neurodevelopmental disorders and different forms of epilepsy.^8,33^ Mutations in *CHRNA4* and *CHRNB2* genes, that encode the α4 and β2 subunits of nAChR, are typically associated with autosomal dominant nocturnal frontal lobe epilepsy, and a recent study found an association between mutations in the gene *CHRNA2*, which encodes the α2 subunit of nAChRs, and the benign familial infantile seizure phenotype.^34^ In addition, α7 subunit-containing AChRs are also essential regulators of seizure susceptibility.^35^ Preliminary results suggest that CRLD-1 is also required for neuronal AChR biosynthesis in *C. elegans* and that inactivation of *CRELD1* in SH-SY5Y neuronal cells impairs the expression of neuronal AChRs. Therefore, we can also hypothesize that specific *CRELD1* compound variants affect the expression of some subtypes of AChRs and ultimately contribute to the neurodevelopmental and epileptic phenotypes observed in our patient.

In addition to its involvement in AChR biosynthesis, CRELD1 likely supports additional functions that may account for the multisystemic phenotype of the patient. Recent studies of gene expression variance in large human cohorts combined with mouse models defined CRELD1 as a key modulator of immune system homeostasis.^17^ CRELD has also been shown to be required for ER-mitochondrial contact dynamics. These enable the transfer of phospholipids, which modulate respiratory complex I activity and promote the generation of hydrogen peroxide as a signalling molecule for dopaminergic neuron function in *Drosophila*.^19^ In *CRELD* fly mutants, the resulting low-hydrogen peroxide levels are associated with disturbed neuronal activity and impaired locomotion. A similar mechanism may be altered in our patient and explain the neurological symptoms.

In conclusion, the *CRELD1* gene can be considered as a candidate for undiagnosed neurodevelopmental disorders, especially when associated with arthrogryposis and epilepsy. If *CRELD1* variants become systematically screened for in the future, variant modelling in invertebrates will provide an interesting means to evaluate variants of unknown significance. A comprehensive analysis of CRELD1 substrates and a further analysis of its physiological functions will potentially identify therapeutic targets.

## Supplementary Material

fcaf326_Supplementary_Data

## Data Availability

The data supporting the findings of this study are available from the corresponding author upon reasonable request.
