# Supplementary material for: Biallelic CRELD1 variants cause severe muscle weakness and infantile epilepsy
Source: Brain Commun. 2025 Sep 3;7(5):fcaf326. doi: 10.1093/braincomms/fcaf326 (PMC12448699; doi:10.1093/braincomms/fcaf326)
Supplement: fcaf326_Supplementary_Data [file fcaf326_Supplementary_Data.pdf]

SUPPLEMENTARY MATERIAL & METHODS

Supplementary Table 1 List of strains and relative genotypes used in this study.

STRAIN	Genotype
N2	
EN298	crld-1(kr298::GFP)
EN308	crld-1a(kr308::GFP)
EN297	crld-1(kr297::HySOG)
EN50584	crld-1a(kr308kr584[C187Y])
EN50585	crld-1a(kr308kr585[C187Y])
EN50586	crld-1a(kr308kr586[C187Y])
EN50587	crld-1a(kr308kr587[C228Y])
EN50588	crld-1a(kr308kr588[C228Y])
EN50589	crld-1a(kr308kr589[C228Y])
EN50871	crld-1(kr298kr871[I257Rfs*25])
EN50872	crld-1(kr298kr872[I257Rfs*25])
EN50873	crld-1(kr298kr873[I257Rfs*25])
EN50874	crld-1a(kr308kr874[I257Rfs*25])
EN50875	crld-1a(kr308kr875[I257Rfs*25])
EN50876	crld-1a(kr308kr876[I257Rfs*25])
EN7980	krSi144(unc-17-cla1-BFP); crld-1(kr297::HySOG)
EN208	unc-29(kr208::TagRFP-T)
EN8607	unc-29(kr208::TagRFP-T);crld-1a(kr308kr586[C187Y])
EN8606	unc-29(kr208::TagRFP-T);crld-1a(kr308kr587[C228Y])
EN8662	unc-29(kr208::TagRFP-T);krSi144(unc-17-cla-1-BFP); crld-1(kr297::HySOG)
EN5780	unc-29(kr208::TagRFP-T); crld-1(kr298::GFP)
EN9598	unc29(kr208::TagRFPT);krSi144(unc-17-cla-1-BFP); crld1(kr298kr871[I257Rfs*25])
EN9599	krSi144(unc-17-cla-1BFP; crld1(kr298kr871[I257Rfs*25])

Supplementary Table 2: List of single strand oligonucleotides (DNA and RNA) for CRISPR/Cas9 genome engineering and genotyping primer combinations for *C. elegans* mutants.

	Sequence (5' to 3')	Description
crRNA MD342	/AltR1/rArA rUrArC rUrCrG rArUrA rUrCrA rCrArG rUrArU rGrUrU rUrUrA rGrArG rCrUrA rUrGrC rU/AltR2/	Use to generate CRLD-1(C187Y)
oMD350	GCCGCGAAGGCAGTGGAAAGTGTAAATGTGAGACTGGATATACTGGcAAcCTcTaCCGcTACTGcGAcATtGAaTAcTTtGAAGAATCTCGGACAGTGCAGGGTG	Repair template containing the C187Y mutation (cursive and blue) and other silent mutations (cursive)
oMD343<br>oMD341	GAAATCTATGCCGATACTGTGATATCG<br>CTTACATTCACACTTGAACGATCCGAC	809bp PCR product in not engineered animals
oMD347<br>oMD341	GcAAcCTcTaCCGcTACTGcGAcATtG<br>CTTACATTCACACTTGAACGATCCGAC	809bp PCR product in engineered animals
crRNA MD336	/AltR1/rGrG rArUrG rCrUrC rArArA rArUrG rUrArA rArArA rGrUrU rUrUrA rGrArG rCrUrA rUrGrC rU/AltR2/	Use to generate CRLD-1(C228Y)
oMD340	cagAATGCCACGAGGGCTGCCTTGGAGTTTGCTCATCGGAGAGCTCAAAAGGcTGtTCcAAgTacAAgAAcGGaTGGAAATTGACAGAAGAAGGGTGCGCAGgtatg	Repair template containing the C228Y mutation (cursive and blue) and other silent mutations (cursive)
oMD338<br>oMD341	GGATGCTCAAAATGTAAAAATGGGTGG<br>CTTACATTCACACTTGAACGATCCGAC	631bp PCR product in not engineered animals
oMD339<br>oMD341	GGcTGtTCcAAgTacAAgAAcGGaTGG<br>CTTACATTCACACTTGAACGATCCGAC	631bp PCR product in not engineered animals
oMD352<br>oMD341	GTGTGCTGCCCTGATGGACACT<br>CTTACATTCACACTTGAACGATCCGAC	1042bp PCR product for sequencing verification for both (C187Y) and (C228Y)
oMD352	GTGTGCTGCCCTGATGGACACT	sequencing verification for both (C187Y) and (C228Y)
oMD206	GTGTTTCGGCAATTCTGCA	sequencing verification for both (C187Y) and (C228Y)

crRNA DLG60	TTCGTGCTCTTTTGTGCATG	Use to generate CRLD-1(I257RFs*25)
oDLG184	ccttatttcagACGTCAACGAATGCCAAAACGAGTCaGCATGCACAAAAGAGCACGAAcgtgtgaaaacaccgagggcggttatcgctgcatctgtgccgagggctacaagcagatggaaggcatctgtgtgaTGTGTGAACACTGTCGGATCGTTCAAGTGTGAATG	Repair template containing the I257RFs*25 mutation (cursive and blue)
oDLG185<br>oDLG187	GGTGCGCAGgtatgagaaaag<br>Ccagaagtaaaccagatcatgc	701 bp PCR product in not engineered animals and 773bp PCR product in engineered animals
oDLG185<br>oDLG186	GGTGCGCAGgtatgagaaaag<br>GCACAGATGCAGCGATAACC	586bp PCR product in engineered animals
oDLG185	GGTGCGCAGgtatgagaaaag	sequencing verification for I257RFs*25

Whole exome sequencing.

Whole exome sequencing (WES) was performed on an Illumina NovaSeq 6000 Sequencer (Illumina, USA) with a paired-end 100 bp sequencing protocol following the manufacturer's best-practice protocol. After enrichment with the SureSelect Human All Exon V7 (Agilent, USA) kit a mean coverage of 93-fold was achieved. Please see supplemental Table 3 for details on WES metrics.

Cov 1x	Cov 2x	Cov 10x	Cov 20x	Cov 30x	Cov 100x	Mean Cov	Total reads	ROH_Sum
98.0	97.7	96.1	93.5	89.5	39.3	93.0	102226826	23

Supplementary Table 3: Detailed metrics of Whole Exome Sequencing in our patient with coverage (1x, 2x, 10x, 20x, 30x, 100x, mean), number of total reads and summary of autosomal Runs of Homozygosity (ROH) in megabases (ROH_Sum) of 298 based on ALLEGRO.(1) The following parameter settings were used to extract high quality

SNVs for the ROH calculation: Maximal observed allele frequency <=0.8% in any gnomAD population for ROH calculation; Genotype Quality >=99, as Phred-scaled confidence that genotype assignment is correct, derived from Phred-scaled likelihood of the considered genotypes in the variant record for each sample; Log odds of being a true variant versus being false under the trained Gaussian mixture model >=2 (with GATK according to Mckenna et al [18]); Quality by Depth>=6; Mapping Quality>=60; Phred-scaled p-value using Fishers exact test to detect strand bias<=20; Z-score from Wilcoxon rank sum test of Alternate vs. Reference Alleles read mapping qualities>=-2.5; Rank Sum Test for relative positioning of Reference versus Alternate alleles within reads >=-2.5.

The sequencing data was analyzed using the Varbank2 pipeline of the Cologne Center for Genomics (https://varbank.ccg.uni-koeln.de/varbank2). Reads were aligned to the GRCh38 human reference genome (without alternate contigs) using BWA-mem (version 0.7.15) (2). Subsequently, duplicates were marked using Picard (version 2.1.1) (http://broadinstitute.github.io/picard/) followed by base quality score recalibration and local indel realignment using GATK (version 3.6) (3). For SNP/Indel calling, three callers were used: GATK HaplotypeCaller (version 3.6) (3), samtools (version 1.6) (4), and platypus (version 0.8.1) (5). Variants called by GATK HaplotypeCaller underwent variant quality score recalibration with GATK (version 3.6).

All variants from the different callers were merged for each sample individually and annotated using in-house-developed scripts. A comprehensive pathogenicity rank score (MedPred) was calculated as median of all pathogenicity rank scores obtained from the dbNSFP database (https://sites.google.com/site/jpopgen/dbNSFP): POLYPHEN2_HDIV and _HVAR (6), SIFT (7), RVIS (8), LRT, MutationTaster, Mutation Assessor, FATHMM, PROVEAN, VEST3, MetaSVM, MetaLR, M-CAP,

REVEL, MutPred 1.2, CADD, DANN, FATHMM-MKL, Eigen-PC, GenoCanyon, fitCons_integr, fitCons_GM12878, fitCons_H1-hESC, fitCons_HUVEC, Gerp++_RS (9), phyloP100way_vert, phyloP20way_mamm, phastCons100way_vert, phastCons20way_mamm, SiPhy_29way, ADA_score, and RF_score. The population allele frequency was calculated as the maximal observed frequency of the variation in any of the reported sub-populations of gnomAD (https://gnomad.broadinstitute.org/). Additionally, our inhouse variant frequency of over 8000 NGS datasets was calculated.

Splice effect prediction was performed for GT-AG-U2 splice sites with MaxEntScan (10) using the Maximum Entropy Model and for U2/U12 splice sites and U12 branch points with Position Weight Matrix (11). The functional interpretation identified the most probable intron types. Filtering was then based on the score change in percent.

CNV calling was performed using XHMM (version 1.0) (12), CoNIFER (version 0.2.2) (13), and ExomeDepth (version 1.1.10) (14), using an in-house collection of 70 WES data sets as a reference. The variant lists from all callers were merged and annotated using dbnsfp (version 3.4a).

Variant filtering.

Single nucleotide variants and short INDELs with an allele frequency of >0,1% in gnomAD database (v2.1.1), >0,2% in our in-house database and all intronic variants with a distance >20bp from the intron/exon border were excluded. The evaluation of the pathogenicity of missense variants was performed using the inhouse scripted MedPred score as combination of multiple in silico prediction tools. Missense variants with a score of <0,4 were considered to have a low probability for pathogenicity. Additionally, data from various public databases was utilized to see the distribution of

genetic variants in healthy probands: dbSNP, (15); ENSEMBL, (16); commercial HGMD professional database, (17) and ClinVar (www.ncbi.nlm.nih.gov/clinvar/). The resulting variants were evaluated according to different inheritance patterns. Taking the non-consanguineous parents and low ROH score into account we primarily filtered for compound-heterozygous autosomal recessive and autosomal dominant variants but also considered other theoretically possible inheritance patterns.

Sanger sequencing.

PCR primer pairs were designed from genomic DNA to amplify and sequence CRELD1 variants in Exon 8 (5′-TGTGGAGCTGACCAATTCTG-3′ and 5′-AACATTCCACCCTTCCTTCC-3′) and Exon 9 (5′-TGAAGAGGCTGGAATATGGG-3′ and 5′-TGATTCCCTTTGTACCCTGG-3′). PCR amplification was carried out using 0.6 U DNA Polymerase (OneTaq), 200 µM dNTP-Mix, 0.25 µM of each primer and 20 ng DNA. After an initial cycle of denaturation at 94°C for 30 sec, 35 cycles were performed consisting in denaturation at 94°C for 20 sec., annealing at 59°C for 20 sec. and extension at 68°C for 30 sec., and final extension 5 min at 68°C, on an ABI9700 Thermal Cycler. PCR products were then purified and sequenced using the forward primers (seqlab). The obtained DNA sequences were compared with published sequences (BLAST, NCBI).

Sequence alignment

Sequence alignment was performed using the promals 3D software with consensus level 0.8 (http://prodata.swmed.edu/promals3d/) DOI: 10.1093/nar/gkn322. Consensus amino acid symbols in Promals3D:

conserved amino acid residues: bold and uppercase letters (such as G);

aliphatic residues (I, V, L): *l*

aromatic residues (Y, H, W, F): @

hydrophobic residues (W, F, Y, M, L, I, V, A, C, T, H): *h*

alcohol residues (S, T): o

polar residues (D, E, H, K, N, Q, R, S, T): p

tiny residues (A, G, C, S): t

small residues (A, G, C, S, V, N, D, T, P): s

bulky residues (E, F, I, K, L, M, Q, R, W, Y): b

positively charged residues (K, R, H): +

negatively charged residues (D, E): -

charged (D, E, K, R, H): c

The alignment was performed using the following sequences:

>NP_001070883.2 protein disulfide isomerase CRELD1 isoform 2 precursor [Homo sapiens]

>NP_598691.1 protein disulfide isomerase Creld1 precursor [Mus musculus]

>NP_001014851.3 protein disulfide isomerase CRELD1 precursor [Bos taurus]

>XP_014985745.1 cysteine-rich with EGF-like domain protein 1 isoform X1 [Macaca mulatta]

>XP_022262103.1 protein disulfide isomerase CRELD1 [Canis lupus familiaris]

>XP_015148835.2 cysteine-rich with EGF-like domain protein 1 [Gallus gallus]

>XP_014464393.2 cysteine-rich with EGF-like domain protein 1, partial [Alligator mississippiensis]

>XP_012817147.1 protein disulfide isomerase CRELD1 [Xenopus tropicalis]

>XP_032893048.1 protein disulfide isomerase CRELD1 [Amblyraja radiata]

>XP_009302639.1 protein disulfide isomerase CRELD1 [Danio rerio]

REFERENCES OF SUPPLEMENTARY MATERIAL & METHODS

1. Gudbjartsson DF, Jonasson K, Frigge ML, and Kong A. Allegro, a new computer program for multipoint linkage analysis. *Nature genetics.* 2000;25(1):12-3.
2. Li H, and Durbin R. Fast and accurate short read alignment with Burrows-Wheeler transform. *Bioinformatics (Oxford, England).* 2009;25(14):1754-60.
3. McKenna A, Hanna M, Banks E, Sivachenko A, Cibulskis K, Kernytsky A, et al. The Genome Analysis Toolkit: a MapReduce framework for analyzing next-generation DNA sequencing data. *Genome research.* 2010;20(9):1297-303.
4. Li H, Handsaker B, Wysoker A, Fennell T, Ruan J, Homer N, et al. The Sequence Alignment/Map format and SAMtools. *Bioinformatics (Oxford, England).* 2009;25(16):2078-9.

5. Rimmer A, Phan H, Mathieson I, Iqbal Z, Twigg SRF, Wilkie AOM, et al. Integrating mapping-, assembly- and haplotype-based approaches for calling variants in clinical sequencing applications. *Nature genetics.* 2014;46(8):912-8.
6. Adzhubei IA, Schmidt S, Peshkin L, Ramensky VE, Gerasimova A, Bork P, et al. A method and server for predicting damaging missense mutations. *Nature methods.* 2010;7(4):248-9.
7. Kumar P, Henikoff S, and Ng PC. Predicting the effects of coding non-synonymous variants on protein function using the SIFT algorithm. *Nature protocols.* 2009;4(7):1073-81.
8. Petrovski S, Wang Q, Heinzen EL, Allen AS, and Goldstein DB. Genic intolerance to functional variation and the interpretation of personal genomes. *PLoS genetics.* 2013;9(8):e1003709.
9. Davydov EV, Goode DL, Sirota M, Cooper GM, Sidow A, and Batzoglou S. Identifying a high fraction of the human genome to be under selective constraint using GERP++. *PLoS computational biology.* 2010;6(12):e1001025.
10. Yeo G, and Burge CB. Maximum entropy modeling of short sequence motifs with applications to RNA splicing signals. *Journal of computational biology : a journal of computational molecular cell biology.* 2004;11(2-3):377-94.
11. Sheth N, Roca X, Hastings ML, Roeder T, Krainer AR, and Sachidanandam R. Comprehensive splice-site analysis using comparative genomics. *Nucleic acids research.* 2006;34(14):3955-67.
12. Fromer M, and Purcell SM. Using XHMM Software to Detect Copy Number Variation in Whole-Exome Sequencing Data. *Current protocols in human genetics.* 2014;81:7.23.1-1.
13. Krumm N, Sudmant PH, Ko A, O'Roak BJ, Malig M, Coe BP, et al. Copy number variation detection and genotyping from exome sequence data. *Genome research.* 2012;22(8):1525-32.
14. Plagnol V, Curtis J, Epstein M, Mok KY, Stebbings E, Grigoriadou S, et al. A robust model for read count data in exome sequencing experiments and implications for copy number variant calling. *Bioinformatics (Oxford, England).* 2012;28(21):2747-54.
15. Sherry ST, Ward MH, Kholodov M, Baker J, Phan L, Smigielski EM, et al. dbSNP: the NCBI database of genetic variation. *Nucleic acids research.* 2001;29(1):308-11.
16. Flicek P, Amode MR, Barrell D, Beal K, Billis K, Brent S, et al. Ensembl 2014. *Nucleic acids research.* 2014;42(Database issue):D749-55.
17. Stenson PD, Ball EV, Howells K, Phillips AD, Mort M, and Cooper DN. The Human Gene Mutation Database: providing a comprehensive central mutation database for molecular diagnostics and personalized genomics. *Human genomics.* 2009;4(2):69-72.

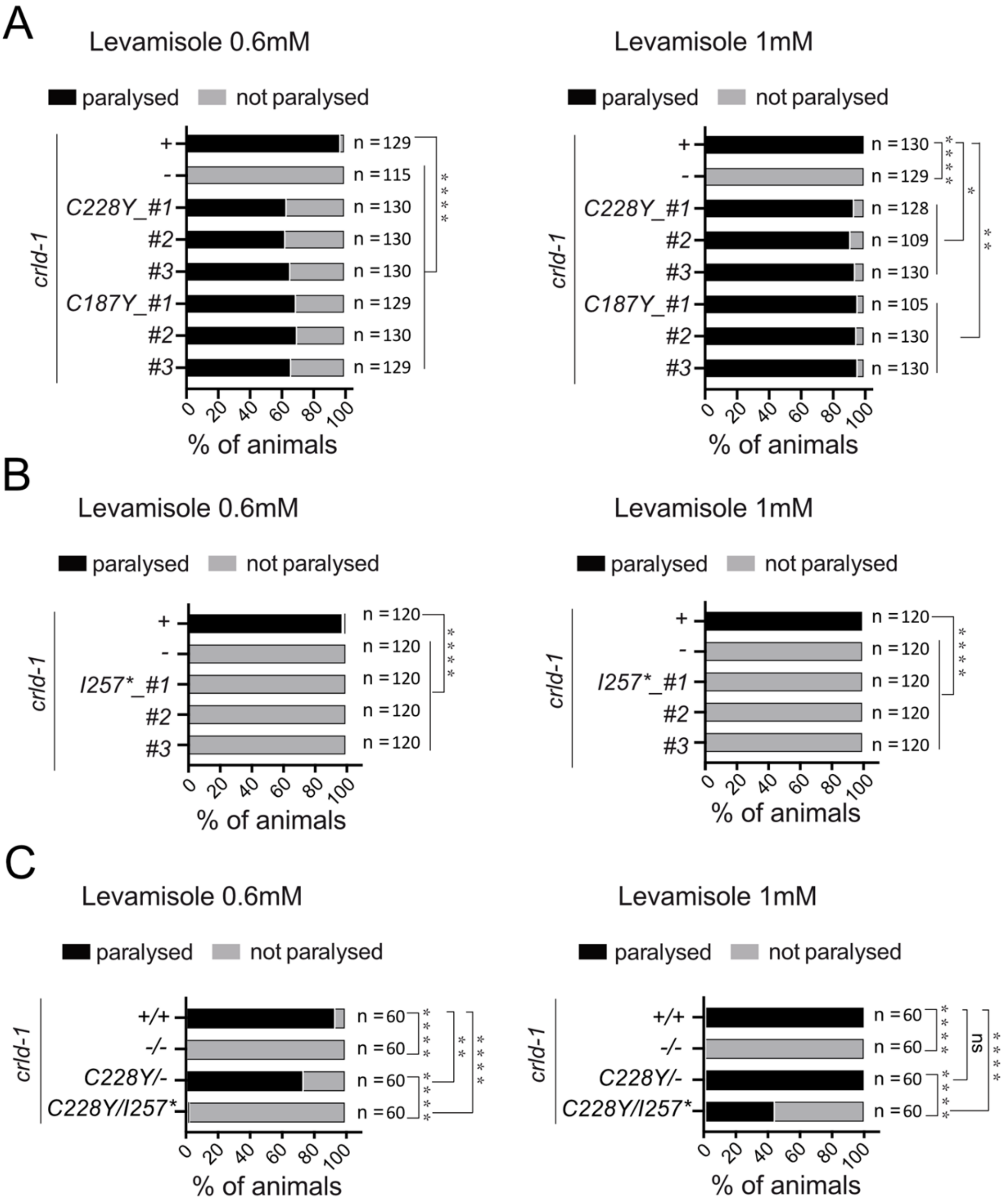

Supplemental Figure 1. Patient's CRELD1 variants affect L-AChR expression to different extents based on sensitivity to levamisole. Levamisole test on both wild types (indicated as +), *crld-1* knock outs (indicated as -), *crld-1a* Cys228Tyr, Cys187Tyr and Ile257Argfs*25 knock ins (indicated as *C228Y*, *C187Y* and *I257**, respectively). For each knock in expressing the patient mutations were generated by CRISPR and tested on levamisole 3 independent lines (indicated as #1, #2, #3).

Experiments were repeated six times, *p<0.05, **p<0.01 and ****p<0.0001, after Bonferroni correction, Fisher exact probability test (Panels A and B). Levamisole test on both wild types (indicated as +/+), *crld-1* knock outs (indicated as -/-) and *crld-1* compound heterozygous mutations obtained by crossing *crld-1 Cys228Tyr* with either *crld-1* knock outs or *crld-1 Ile257Argfs*25* knock ins (indicated as *C228Y/-* and *C228Y/I257*,* respectively). *C228Y/I257** animals are largely resistant to levamisole, whereas *C228Y/-* animals show a more wild-type-like response. Experiments were repeated three times. ns = not significant p = 1 for *C228Y/- on* 1 mM levamisole, **p<0.01 and ****p<0.0001, after Bonferroni correction, Fisher exact probability test. Gray bars indicate the % of moving animals after overnight exposure to either 1 mM or 0.6 mM levamisole, and black bars indicate the % of paralyzed animals, the number of animals tested for each genotype is indicated (Panel C).

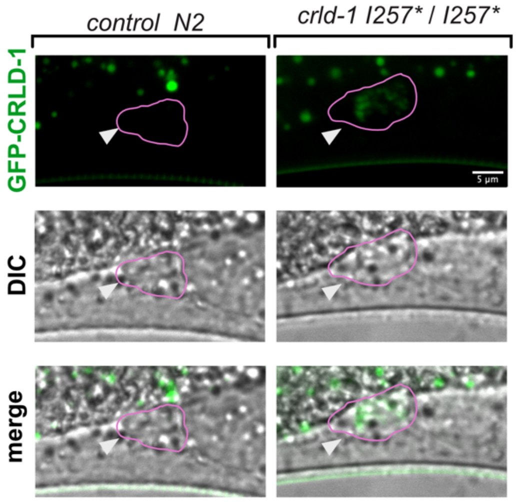

Supplemental Figure 2. CRELD-1 is present in the coelomocytes of *I257 mutants.** Confocal and DIC images of the ventral pair of coelomocytes located near the head in both N2 wild-type and *I257** mutant animals. Coelomocytes are outlined in pink and indicated with arrowheads. In *I257** mutants, GFP-CRLD-1 signal is detectable in the coelomocytes. Outside the coelomocytes, the GFP signal corresponds to autofluorescent intestinal granules in both N2 and *I257** animals. Scale bar = 5 µm.

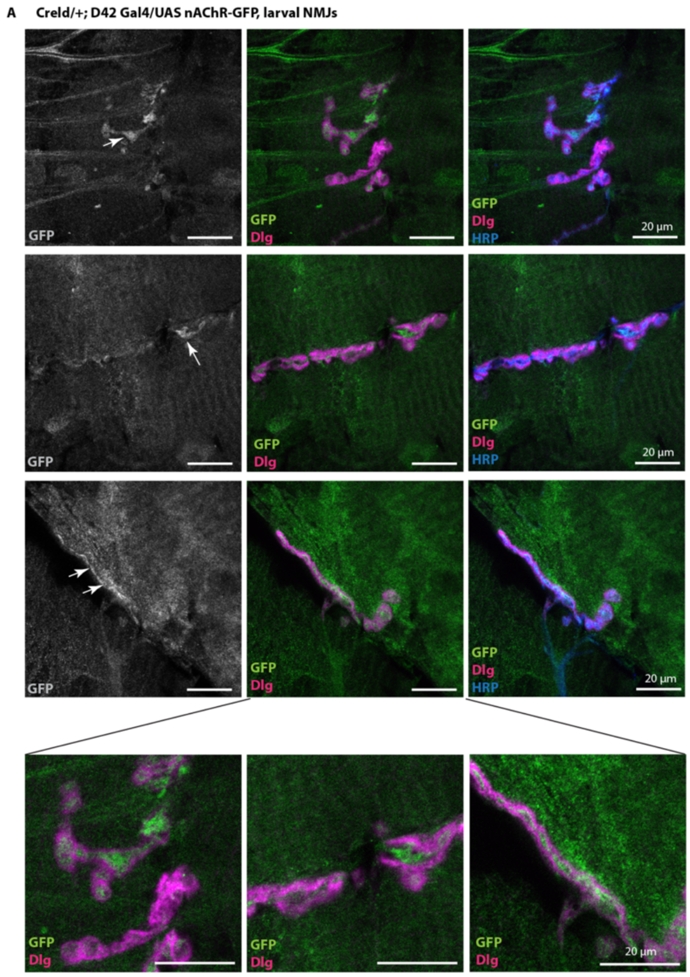

Supplemental Figure 3A. Heterozygous control: nicotinic Acetylcholine receptor marked with GFP (nAChR-GFP) expressed in *Drosophila* larval NMJs using the motor neuron Gal4 driver D42-Gal4. Shown are 3 examples of NMJs expressing nAChR-GFP (shown in green, left panel). Arrows indicate expression of nAChR in NMJ boutons. NMJs are marked with antibodies against the synapse protein Discs large (Dlg, shown in magenta, middle panel) and neurons are marked with HRP (shown in blue, merge, right panel). Close-ups are shown in the lower panel. Scale bars as indicated.

B Creld-/-; D42 Gal4/UAS nAChR-GFP, larval NMJs

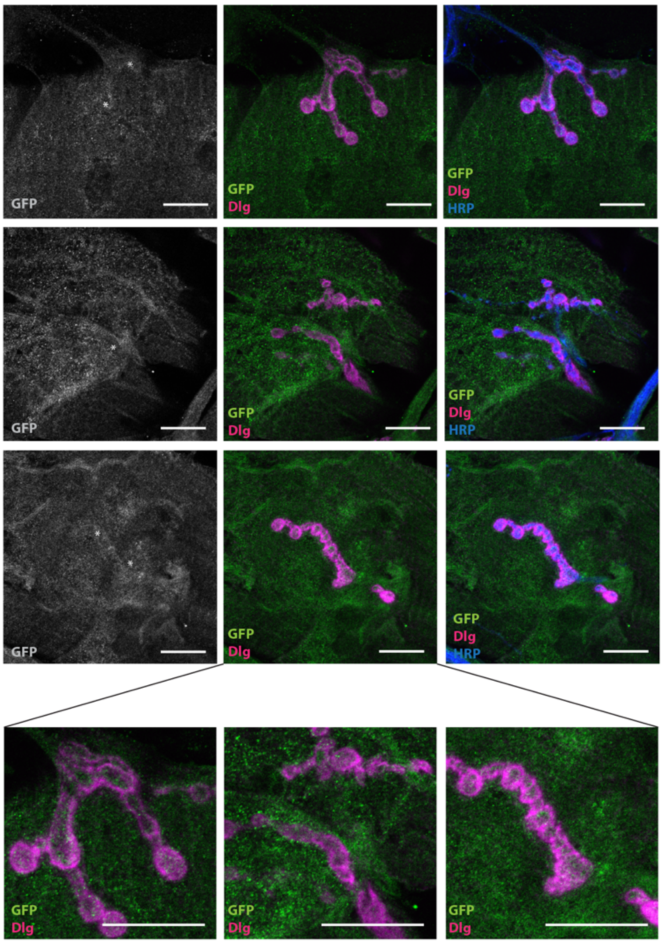

Supplemental Figure 3B. Homozygous *Creld* mutants: nicotinic Acetylcholine receptor marked with GFP (nAChR-GFP) expressed in *Drosophila* larval NMJs using the motor neuron Gal4 driver D42-Gal4. Shown are 3 examples of NMJs expressing nAChR-GFP (shown in green, left panel). Asterisks indicate missing expression of nAChR in NMJ boutons. NMJs are marked with antibodies against the synapse protein Discs large (Dlg, shown in magenta, middle panel) and neurons are marked with HRP (shown in blue, merge, right panel). Close-ups are shown in the lower panel. Scale bars as indicated.

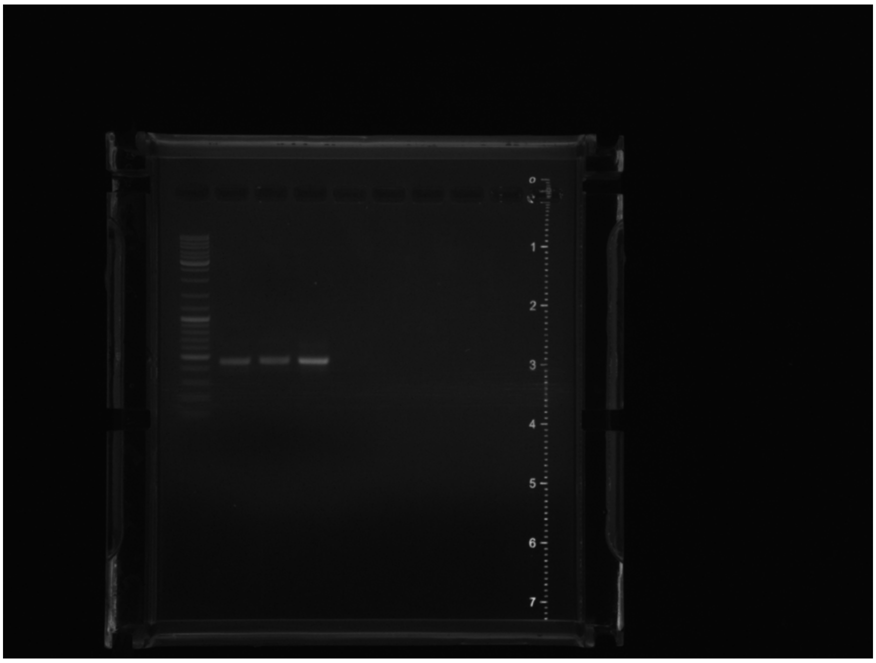

Full-size uncropped blot referred to Figure 2D
